# Efficacy and Safety of Dotinurad in Hyperuricemic Patients With or Without Gout: A Systematic Review and Meta-Analysis of Randomized Controlled Trials

**DOI:** 10.7759/cureus.14428

**Published:** 2021-04-12

**Authors:** Ayman Iqbal, Kinza Iqbal, Eisha Farid, Ali Ishaque, Muhammad Hasanain, Taha Bin Arif, Shajeea Arshad Ali, Sawai Singh Rathore, Mehreen Malik

**Affiliations:** 1 Internal Medicine, Dow University of Health Sciences, Karachi, PAK; 2 Internal Medicine, Dr. Sampurnanand Medical College, Jodhpur, IND; 3 Anesthesiology, Aga Khan University, Karachi, PAK

**Keywords:** dotinurad, fyu-981, selective urate reabsorption inhibitor, urat1 inhibitor, hyperuricemia

## Abstract

Introduction

A systematic review and meta-analysis of the available randomized controlled trials (RCTs) were conducted to investigate the efficacy and safety of dotinurad in hyperuricemic patients with or without gout. Dotinurad is a novel selective urate reabsorption inhibitor (SURI) that increases uric acid excretion by selectively inhibiting urate transporter 1 (URAT1). To the best of our knowledge, this is the first meta-analysis conducted to gauge the efficacy and safety of dotinurad.

Methods

Electronic databases (PubMed, the Cochrane Library, and ClinicalTrials.gov) were searched from inception till March 2, 2021, according to the Preferred Reporting Items for Systematic Review and Meta-Analysis statement. Randomized controlled trials comparing the efficacy and safety of dotinurad with placebo- or active (febuxostat or benzbromarone) control were included. The eligible studies were analyzed with RevMan 5.3 Software (The Nordic Cochrane Centre, Cochrane Collaboration, Copenhagen).

Results

Four eligible studies, consisting of 684 hyperuricemic patients were included. The number of patients who achieved serum uric acid (sUA) levels ≤ 6.0 mg/dl favoured dotinurad 1 mg group as compared to placebo group (risk ratio {RR} = 39.27, 95% onfidence interval {CI}, 5.59 to 275.65; p = 0.0002), dotinurad 2 mg group compared with placebo group (RR = 45.36, 95% CI, 6.48 to 317.38; p= 0.0001), and dotinurad 4 mg group compared with placebo group (RR = 54.16, 95% CI, 7.76 to 377.77; p < 0.0001). Conversely, there was no significant difference in the number of patients who achieved the target sUA levels between dotinurad 2 mg and active control (RR = 1.00, 95% CI, 0.92 to 1.08; p = 0.91). Moreover, the percentage change in sUA levels from baseline to final visit favoured dotinurad 1 mg vs. placebo ((RR = 36.51, 95% CI, 33.00 to 40.02; p < 0.00001), dotinurad 2 mg vs. placebo (RR = 46.70, 95% CI, 42.53 to 50.87; p < 0.00001), and dotinurad 4 mg vs. placebo (RR = 63.84, 95% CI, 60.51 to 67.16; p < 0.00001), while no significant difference was seen in dotinurad 2 mg vs. active control (RR = -0.08, 95% CI, -4.27 to 4.11; p= 0.97). Compared with active or placebo control, dotinurad 2 mg showed no significant difference in the number of events of gouty arthritis (RR= 1.31, 95% CI, 0.47 to 3.71; p = 0.60), the number patients with adverse events (RR = 1.09, 95% CI, 0.91 to 1.30; p = 0.36), and the number of patients who experienced adverse drug reactions (RR = 1.00, 95% CI, 0.68 to 1.47; p = 0.99).

Conclusion

Dotinurad shows significant improvement in serum uric acid levels in hyperuricemic individuals with or without gout. Its urate-lowering effect is comparable to the commonly available anti-hyperuricemic agents. Moreover, it is effective at doses 1 mg, 2 mg, and 4 mg and well-tolerated at a dose of 2 mg.

## Introduction

Hyperuricemia, defined as a serum uric acid level > 7 mg/dl, is associated with an increased risk of urate deposition diseases, namely gout, uric acid nephrolithiasis, and urate neuropathy [1]. Additionally, an increased prevalence of chronic kidney disease (CKD), diabetes mellitus (DM), and hypertension (HTN) exist in patients with increased serum uric acid levels [2]. The global burden of hyperuricemia and gout is rising, projecting that gout mortality may increase by 55% in 2060 [3].

The third edition of Japanese guidelines classifies hyperuricemia as under-excretion, combined, and renal load type [2]. The renal load type encompasses the overproduction and extra-renal under-excretion hyperuricemia [2]. The latter is due to reduced intestinal urate excretion caused by ATP-binding cassette transporter, sub-family G, member 2 (ABCG2) dysfunction [4]. ABCG2 dysfunction hinders the usually occurring 30% intestinal urate excretion, increasing renal load [5].

Moreover, 85-90% of hyperuricemic patients are known to have asymptomatic hyperuricemia [6]. These asymptomatic patients do not exhibit clinical features of gout [6]. Japanese guidelines for the management of hyperuricemia and gout recommend drug therapy for asymptomatic hyperuricemic patients with comorbidities like DM, HTN, and CKD when serum uric acid level is ≥ 8 mg/dl [7]. The pharmacological therapy aims to reduce and maintain a serum uric acid level at ≤ 6 mg/dl [7].

Presently, allopurinol and febuxostat, both xanthine oxidase inhibitors, are employed for treating overproduction hyperuricemia [8]. Whereas uricosuric drugs, such as probenecid and benzbromarone, are recommended for the under-excretion type [8]. Allopurinol and febuxostat inhibit xanthine oxidase, reducing the conversion of xanthine to uric acid [1]. However, despite being the first-line urate-lowering drug, allopurinol carries a risk of Stevens-Johnson syndrome, toxic epidermal necrolysis, and hypersensitivity syndrome of allopurinol [7]. While severe hepatotoxicity, including fulminant hepatitis, is associated with benzbromarone [8]. Similarly, probenecid use has also shown adverse drug reactions (ADRs) such as urate nephropathy, especially in patients with renal impairment [1]. Furthermore, severe acute kidney injury occurred on high dose monotherapy with lesinurad, the newly approved uricosuric agent [8].

Hence, in light of the notably higher prevalence of under-excretion hyperuricemia than the overproduction type, there is a pressing need for safer and more effective uricosuric drugs for the treatment of under-excretion hyperuricemic patients [9]. Dotinurad, a novel selective urate reabsorption inhibitor (SURI), increases uric acid excretion by the kidneys by selectively inhibiting urate transporter 1 (URAT1), the protein responsible for the reabsorption of uric acid, in the proximal renal tubules [10]. No inhibitory effect is exerted on organic anion transporter (OAT) 1 and 3, and ABCG2, the transporters responsible for renal and intestinal uric acid secretion, respectively [10]. However, no conclusive evidence on the efficacy and safety of dotinurad is available. A recent review assessed the safety and effectiveness of dotinurad, but no pooled analysis was performed [9]. Therefore, we conducted a systematic review and meta-analysis of the available randomized controlled trials (RCTs) to investigate the efficacy and safety of dotinurad in hyperuricemic patients with or without gout as compared to placebo and active-control (febuxostat and benzbromarone).

## Materials and methods

We followed Cochrane Collaboration guidelines and Preferred Reporting Items for Systematic Reviews and Meta-Analysis (PRISMA) in conducting this systematic review and meta-analysis [11,12].

Data sources and search strategy

Two authors independently used PubMed, the Cochrane Library, and ClinicalTrials.gov to conduct a rigorous literature search from inception till March 2, 2021. The key search terms included were, “Dotinurad”, “FYU-981”, “Selective urate reabsorption inhibitor”, “URAT1 inhibitor”, and “hyperuricemia”. There was no restriction of language or year of publication. After removing the duplicates and screening the titles and abstracts, full-texts of the retrieved articles were reviewed. We also considered grey literature and bibliographies of relevant articles. Subsequently, only those studies that met our predefined inclusion criteria were included.

Inclusion criteria and exclusion criteria

Studies were included if they met the following inclusion criteria: (1) randomized controlled trials that evaluated the efficacy and safety of dotinurad versus placebo- or active control, (2) trials must have hyperuricemic patients with a serum uric acid (sUA) level at least ≤ 6.0 mg/dl during the run-in period, and (3) trials must clearly mention drug dosage and report at least one of the desired outcomes. The exclusion criteria were pre-determined as follows: (1) duplicate publications, (2) trials without a placebo- or an active control group, (3) studies with patients ≤ 18 years or sample size of ≤ 5 patients, and (4) case reports, letters, reviews, and posters. 

Data extraction and quality assessment

The assessment of eligibility and data extraction were undertaken by two independent investigators and any disagreements were resolved by discussion with a third reviewer. The following data were entered into a standard Excel form: the name of the first author, publication year, study design, inclusion/exclusion criteria, sample sizes and treatment protocols of intervention and control groups, and relevant efficacy and safety outcomes. The Cochrane risk of bias tool was deployed by two researchers independently to appraise each included trial [13]. Any disagreement was resolved after discussion with a third researcher until a consensus was reached. The following domains were assessed: (1) random sequence generation, (2) allocation concealment, (3) selective reporting, (4) incomplete outcome data, (5) blinding of participants/personnel, outcome assessment, and (6) any other bias. To summarize the findings, the risk of bias table was made classifying the grade of bias as high-, unclear-, or low-risk.

Outcome measures

The primary outcome of interest was the number of patients who achieved the sUA level of ≤ 6 mg/dl at the final visit. Other desired outcomes included the percentage of change in sUA levels from baseline to the final visit, the number of events of gouty arthritis, the number of patients who experienced adverse events (AE), and the number of patients who experienced adverse drug reactions (ADRs).

Statistical analyses

Data analysis was performed using Review Manager (RevMan) 5.3 (The Nordic Cochrane Centre, Copenhagen, The Cochrane Collaboration, 2014) [14]. Dichotomous outcomes were pooled using risk ratio (RR) with Mantel-Haenszel random-effects model. Continuous outcomes were analyzed by mean difference using the inverse variance random-effects model. Additionally, 95% confidence intervals (CI) were reported for each outcome. Heterogeneity was evaluated using the Cochrane Q statistics with I2 > 75% regarded as a high degree of heterogeneity [15]. For all analyses, probability values < 0.05 were considered to be statistically significant. Publication bias was not explored as the number of studies included was less than 10.

## Results

**Figure 1:**
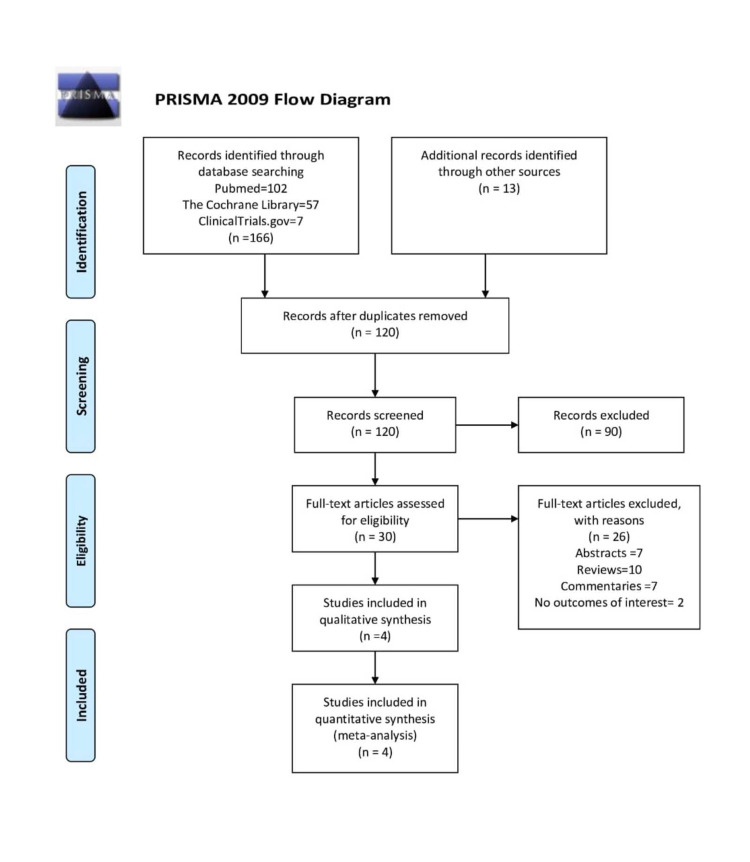
PRISMA flowchart of the study inclusion criteria PRISMA: Preferred Reporting Items for Systematic Reviews and Meta-Analyses

The initial database searches yielded 166 records. After removing duplicates and screening titles and abstracts, 30 full-text articles were reviewed. Finally, four RCTs (684 patients) were eligible for inclusion in the study (Figure 1) [2,7,8,10].

Table 1 summarizes the baseline characteristics of the included studies.

**Table 1:** Baseline characteristics of included trials evaluating the efficacy and safety of dotinurad compared to placebo or active control HTN: hypertension, DM: diabetes mellitus, MS: metabolic syndrome; SD: standard deviation

First author, year	Clinical trials gov ID	Study design	Treatment duration	Main inclusion criteria	Study group	Patients (n)	Age (years), mean ± SD	Males, n (%)
Study 1: Hosoya, 2019 [2]	NCT02344862	Exploratory phase 2, randomized, multicenter, double-blind, placebo-controlled, parallel-group, dose-escalation study	8 weeks	Serum uric acid level during the run-in period ≥ 7.0 mg/dl (patients with a history of gouty arthritis or gouty tophus), ≥ 8.0 mg/dl (patients with asymptomatic hyperuricemia with HTN, DM, and/or MS), or ≥ 9.0 mg/dl (asymptomatic hyperuricemia without above-mentioned comorbidities)	Dotinurad - 1 mg	20	52.7 ± 9.4	20 (100)
					Dotinurad - 2 mg	19	50.1 ± 8.3	19 (100)
					Dotinurad - 4 mg	21	51.2 ± 7.4	21 (100)
					Placebo	20	48.8 ± 9.7	20 (100)
Study 2: Hosoya, 2019 [7]	NCT02416167	Late phase 2, randomized, multicenter, double-blind, placebo-controlled, parallel-group, dose-escalation study	12 weeks	Serum uric acid level during the run-in period ≥ 7.0 mg/dl (patients with a history of gouty arthritis or gouty tophus), ≥ 8.0 mg/dl (patients with asymptomatic hyperuricemia with HTN, DM, and/or MS), or ≥ 9.0 mg/dl (asymptomatic hyperuricemia without above-mentioned comorbidities)	Dotinurad - 0.5 mg	39	58.5 ± 12.1	39 (100)
					Dotinurad - 1 mg	42	57.4 ± 12.4	42 (100)
					Dotinurad - 2 mg	39	55.0 ± 13.5	38 (97.4)
					Dotinurad - 4 mg	40	58.0 ± 10.5	39 (97.5)
					Placebo	39	52.8 ± 11.0	38 (97.4)
Study 3: Hosoya, 2020 [8]	NCT03100318	Phase 3, randomized, multicenter, double-blind, parallel-group, dose-escalation, benzbromarone controlled, comparative study	14 weeks	Serum uric acid level during the run-in period ≥ 7.0 mg/dl (patients with a history of gouty arthritis or gouty tophus), ≥ 8.0 mg/dl (patients with asymptomatic hyperuricemia with HTN, DM, and/or MS), or ≥ 9.0 mg/dl (asymptomatic hyperuricemia without above-mentioned comorbidities)	Dotinurad - 2 mg	102	55.0 ± 10.3	100 (98.0)
					Benzbromarone - 50 mg	98	54.7 ± 10.4	98 (100)
Study 4: Hosoya, 2020 [10]	NCT03372200	Phase 3, multicenter, randomized, double-blind, Active-controlled, parallel-group, dose-titration study	14 weeks	Serum uric acid level during the run-in period ≥ 7.0 mg/dl (patients with a history of gouty arthritis or gouty tophus), ≥ 8.0 mg/dl (patients with asymptomatic hyperuricemia with HTN, DM, and/or MS), or ≥ 9.0 mg/dl (asymptomatic hyperuricemia without above-mentioned comorbidities)	Dotinurad - 2 mg	99	55.1 ± 10.8	99 (100)
					Febuxostat - 40 mg	100	57.1 ± 10.6	100 (100)

All the included studies assessed the efficacy and safety of dotinurad in hyperuricemic patients with or without gout. Two trials compared dotinurad with placebo, while the rest of the two used active control, i.e, benzbromarone and febuxostat as a comparator [2,7,8,10].

Risk of bias

The risk of bias assessment for randomized controlled trials included in our study was summarized in Table 2. 

**Table 2:** Assessment of methodological quality of included trials

Study	Random sequence generation	Allocation concealment	Selective reporting	Incomplete outcome data	Other bias	Blinding of participants/personnel	Blinding of outcome assessment
Study 1: Hosoya, 2019 [2]	Low-risk	Unclear	Low-risk	Low-risk	Low-risk	Low-risk	Low-risk
Study 2: Hosoya, 2019 [7]	Low-risk	Unclear	Low-risk	Low-risk	Low-risk	Low-risk	Low-risk
Study 3: Hosoya, 2020 [8]	Low-risk	Unclear	Low-risk	Low-risk	Low-risk	Low-risk	Low-risk
Study 4: Hosoya, 2020 [10]	Low-risk	Unclear	Low-risk	Low-risk	Low-risk	Low-risk	Unclear

The risk of bias for random sequence generation was low for all studies, however, it was unclear for allocation concealment [2,7,8,10]. All trials were randomized, double-blind studies [2,7,8,10]. Two trials [2,7] were placebo-controlled, and two trials were active-controlled studies [8,10]. Selective reporting bias was low in all studies [2,7,8,10], while specific information for blinding of outcome assessors was provided for three trials [2,7,8]. No other source of bias was evident in the studies. 

Outcomes

The Number of Patients With an sUA Level ≤ 6.0 mg/dl at the Final Visit

All four RCTs reported the number of patients with an sUA level ≤ 6.0 mg/dl at the final visit with two trials comparing different dosages of dotinurad with placebo, and the other two trials comparing dotinurad 2 mg with benzbromarone and febuxostat [2,7,8,10].

**Figure 2:**
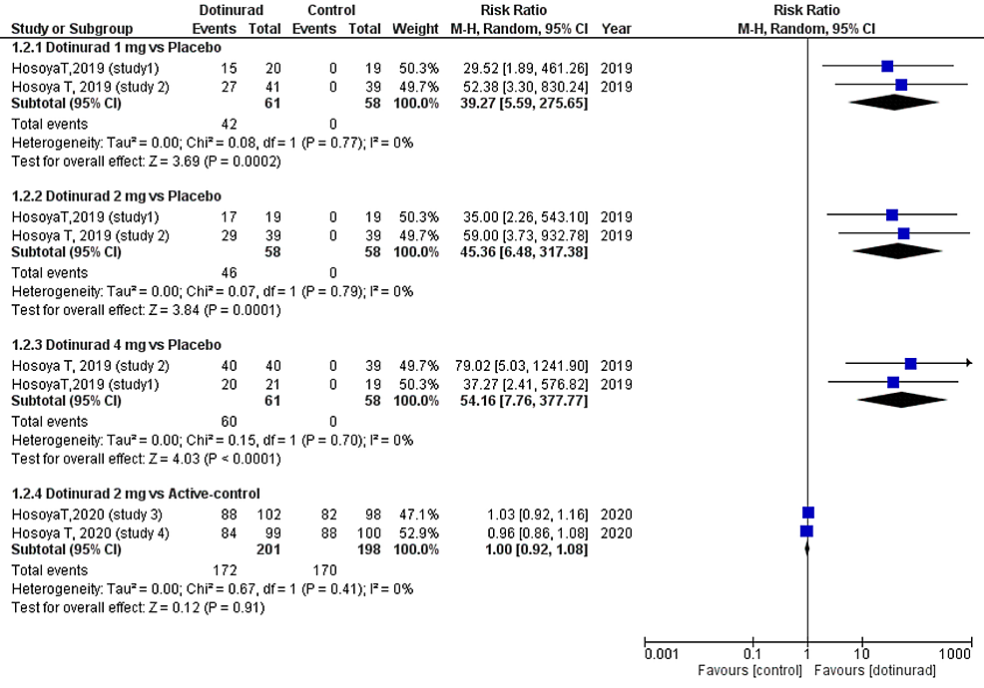
Forest plot comparing the proportion of patients with serum uric acid level < 6.0 mg/dl at the final visit between dotinurad 1, 2, or 4 mg versus placebo or active control Active-control (i.e., febuxostat and benzbromarone) CI: confidence interval; M-H: Mantel-Haenszel; df: degrees of freedom

Figure 2 illustrates the classification of patients into four subgroups according to the dosage of dotinurad (1 mg, 2 mg, and 4 mg) and different control groups (placebo or active). The number of patients who achieved the desired sUA levels favoured dotinurad 1 mg group as compared to placebo group (RR = 39.27, 95% CI, 5.59 to 275.65; p = 0.0002, I2 = 0%), dotinurad 2 mg group compared with placebo group (RR = 45.36, 95% CI, 6.48 to 317.38; p = 0.0001, I2= 0%), and dotinurad 4 mg group compared with placebo group (RR = 54.16, 95% CI, 7.76 to 377.77; p < 0.0001, I2 = 0%) (Figure 2). Conversely, there was no significant difference in the number of patients who achieved the target sUA levels between dotinurad 2 mg and active control (RR = 1.00, 95% CI, 0.92 to 1.08; p = 0.91, I2= 0%) (Figure 2). 

The Percentage Change in sUA Levels From the Baseline to Final Visit

**Figure 3:**
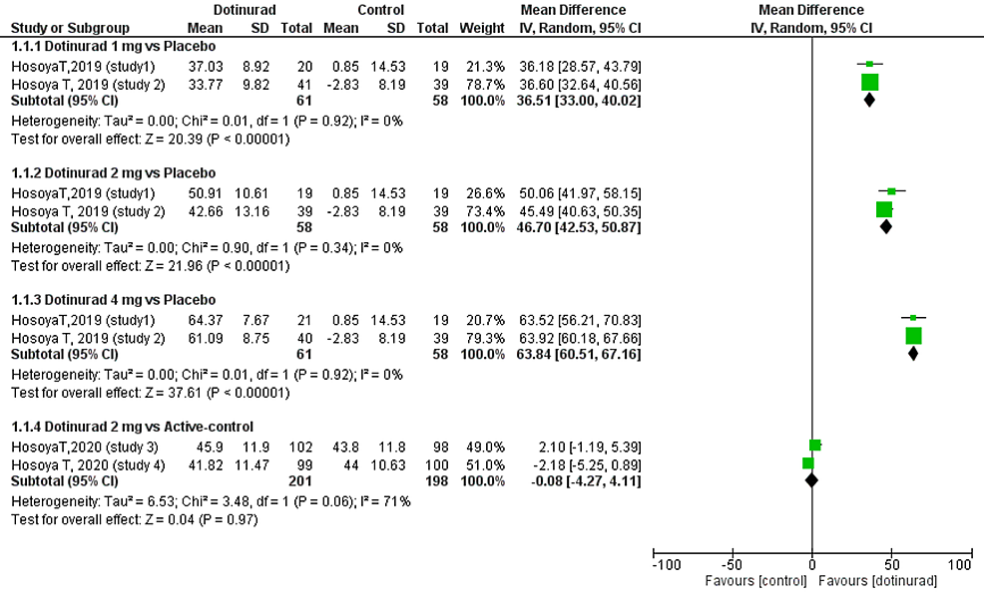
Forest plot comparing the percentage change in serum uric acid level from the baseline to final visit between dotinurad 1, 2, or 4 mg versus placebo or active control Active-control (i.e., febuxostat and benzbromarone); Mean: mean serum uric acid levels SD: standard deviation; CI: confidence interval; IV: inverse variance; df: degrees of freedom

All the evaluated trials measured the percentage change in sUA levels from the baseline to the final visit. Patients were categorized into four subgroups stratified by the dosage of dotinurad (1 mg, 2 mg, and 4 mg) and different control groups (placebo or active), as shown by Figure 3. The percentage change in sUA levels favoured dotinurad 1 mg vs. placebo (RR = 36.51, 95% CI, 33.00 to 40.02; p < 0.00001, I2 = 0%), dotinurad 2 mg vs. placebo (RR = 46.70, 95% CI, 42.53 to 50.87; p < 0.00001, I2 = 0%), and dotinurad 4 mg vs. placebo (RR = 63.84, 95% CI, 60.51 to 67.16; p < 0.00001, I2 = 0%) (Figure 3). However, there was no statistically significant difference in the percentage change of sUA levels in those patients who received dotinurad 2 mg compared with active control (i.e., those who received febuxostat or benzbromarone) (RR = -0.08, 95% CI, - 4.27 to 4.11; p = 0.97, I2= 71%) (Figure 3).

The Number of Events of Gouty Arthritis (Dotinurad 2 mg vs. Control)

**Figure 4:**
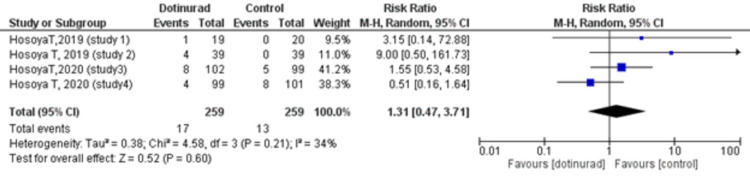
Forest plot comparing the number of events of gouty arthritis between dotinurad 2 mg versus placebo or active control CI: confidence interval; M-H: Mantel-Haenszel; df: degrees of freedom

All included trials investigated the number of events of gouty arthritis. We compared the events of gouty arthritis reported in the dotinurad 2 mg group with the control group. A total of 17 events (6.6%) occurred among 259 patients in the dotinurad 2 mg group versus 13 (5.0%) events among 259 patients in the control group. The pooled estimate showed that there was no statistically significant difference in the number of events of gouty arthritis reported in the dotinurad group 2 mg compared with the control (placebo and active) group (RR = 1.31, 95% CI, 0.47 to 3.71; p = 0.60, I2 = 34%) (Figure 4).

The Number of Patients With Adverse Events (Dotinurad 2 mg vs. Control)

**Figure 5:**
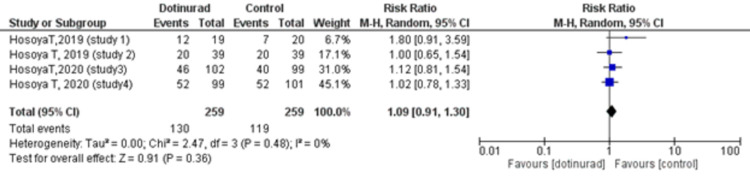
Forest plot comparing the number of patients with adverse events between dotinurad 2 mg versus placebo or active control CI: confidence interval; M-H: Mantel-Haenszel; df: degrees of freedom

All four studies reported the number of patients who experienced AE. We compared this safety outcome between the dotinurad 2 mg group and the control (placebo or active) group. A total of 130 (50.2%) out of 259 patients who received dotinurad 2 mg presented with AE, while 119 (45.9%) out of the 259 patients in the control group reported AE. The pooled estimate revealed no statistically significant association of dotinurad with AE as compared to the control group (RR = 1.09, 95% CI, 0.91 to 1.30; p = 0.36, I2 = 0%) (Figure 5).

The Number of Patients With Adverse Drug Reactions (Dotinurad 2 mg vs. Control)

**Figure 6:**
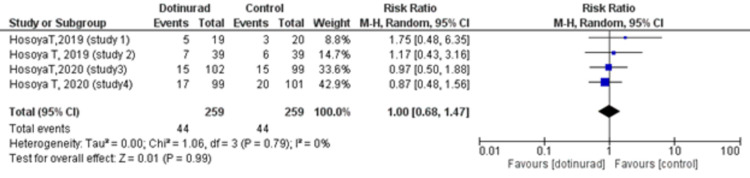
Forest plot comparing the number of patients with adverse drug reactions between dotinurad 2 mg versus placebo or active control CI: confidence interval; M-H: Mantel-Haenszel; df: degrees of freedom

Four RCTs evaluated the number of patients who experienced ADRs. ADRs were reported by an equal number, i.e., 44 (17.0%) out of 259 patients in both the dotinurad and the control group. Therefore, the pooled estimates also showed no statistically significant difference in the number of patients who experienced ADRs between the dotinurad 2 mg group and the control group (RR = 1.00, 95% CI, 0.68 to 1.47; p = 0.99, I2 = 0%) (Figure 6).

## Discussion

To the best of our knowledge, this is the first meta-analysis conducted to gauge the safety and efficacy of the newly developed selective URAT1 inhibitor, dotinurad. When compared with a placebo, dotinurad was found to have a significantly higher efficacy at all doses (1, 2, and 4 mg). Moreover, dotinurad was found to be non-inferior to the already available urate-lowering therapy (ULT), benzbromarone, and febuxostat. Furthermore, we found that dotinurad was not associated with a significant risk of adverse events or adverse drug reactions among hyperuricemic individuals compared with the control (placebo and active).

Uricosuric drugs lower serum uric acid levels by blocking urate reabsorption transporters expressed by the renal tubular epithelial cells, including urate transporter 1 (URAT1) and glucose transporter 9 (GLUT9) [16,17]. However, commonly used uricosuric agents such as probenecid, benzbromarone, and febuxostat have also been known to interact with urate secretion transporters ABCG2, OAT1, and OAT3 [18,19]. The ability of benzbromarone and febuxostat, among other urate-lowering drugs, to inhibit urate secretion activity of ABCG2 has been demonstrated [20]. Considering the role of ABCG2 in both renal and intestinal urate excretion, this could have a somewhat canceling effect on the hypouricemic activity of these drugs [16,20,21]. Furthermore, potent inhibitors of OAT seem to reduce the renal clearance of organic acids e.g., salicylates and methotrexate, resulting in drug-drug interactions [18,22,23]. Given the above, and the finding that URAT1 is the primary protein involved in the reabsorption of urate [23], the attention of new uricosuric agents is focused on selective inhibition of URAT1 to maximize efficacy and minimize unfavorable effects. An example of a SURI, lesinurad was approved for use in the United States and Europe in 2015 and 2016 respectively [22]. Similarly, dotinurad, a novel SURI, was developed for use in hyperuricemic patients in Japan in 2018 [9].

Dotinurad, a phenol derivative, was selected for development from several compounds in a series of studies aimed at countering the unfavorable effects imparted to benzbromarone by its chemical structure [23]. Despite its potent urate-lowering effect, benzbromarone was removed from the market in the USA and several European countries in 2003 due to reports of hepatic impairment [24]. Various mechanisms have been hypothesized for benzbromarone-induced hepatotoxicity. Its chemical structure has been linked to mitochondrial inhibitory activity (MIA) as an explanation for hepatic injury [25]. Hence, dotinurad was developed to retain the beneficial effects of benzbromarone in treating hyperuricemia while simultaneously overcoming any disadvantages related to structural flaws. Dotinurad was shown to have a weaker MIA with half-maximal inhibitory concentration (IC50) being 27 µM compared to 3.1 µM for benzbromarone [23]. Moreover, an in vitro evaluation of URAT1 inhibition showed that dotinurad is five times more potent than benzbromarone (IC50 of 37.2 nM compared to 190 nM for benzbromarone) [23,26]. Furthermore, a study evaluating the effects of uricosuric agents on urate secretion transporters reported that dotinurad did not affect urate secretion activity as opposed to similar drugs, benzbromarone, febuxostat, lesinurad, and probenecid [26]. Therefore, dotinurad possesses an advantage over other urate-lowering drugs in that it has a higher URAT1 selectivity while having no significant effect on the activity of urate secretion transporters [26]. 

Our results showed no significant difference in the efficacy of dotinurad 2 mg in lowering serum urate levels below 6.0 mg/dl when compared to benzbromarone 50 mg (RR = 1.03, 95% CI, 0.92 to 1.16) and febuxostat 40 mg (RR = 0.96, 95% CI, 0.86 to 1.08), demonstrating the non-inferiority of dotinurad to the already available ULT. Moreover, all doses of dotinurad significantly increased the proportion of patients reaching the target serum urate levels when compared to a placebo, (dotinurad 1 mg vs. placebo RR = 39.27, 95% CI, 5.59 to 275.65; p = 0.0002, I2 = 0%), (dotinurad 2 mg vs. placebo RR = 45.36, 95% CI, 6.48 to 317.38; p = 0.0001, I2 = 0%), and (dotinurad 4 mg vs. placebo RR = 54.16, 95% CI, 7.76 to 377.77; p < 0.0001, I2 = 0%).

The safety factors included in our meta-analysis yielded non-significant results. We found no significant difference in the incidence of AEs (dotinurad 2 mg vs. control RR = 1.09, 95% CI, 0.91 to 1.30; p = 0.36, I2 = 0%). With respect to ADRs, 44 events out of a total of 259 were recorded in both the dotinurad 2 mg group and the control group (RR = 1.00, 95% CI, 0.68 to 1.47; p = 0.99, I2 = 0%). Furthermore, among these ADRs were 17 (6.6%) events of gouty arthritis in the treatment group while 13 (5.0%) events were reported in the control group with no statistically significant correlation between the risk of gout flares and the use of dotinurad (2 mg vs. control RR = 1.31, 95% CI, 0.47 to 3.71; p = 0.60, I2 = 34%). Interestingly, a long-term observational study assessing the safety of dotinurad monotherapy demonstrated a decrease in the frequency of gouty arthritis with continued therapy, the incidence being below 1% at 34 and 58 weeks [27]. Despite these findings, a cautious approach is advised due to the tendency of lesinurad, also a SURI, to negatively impact renal function [22]. The renal dysfunction is explained by the increased urinary urate clearance which exacerbates the risk of urinary stone formation in hyperuricemic individuals [28]. Although this risk seems to be ameliorated when lesinurad is used in conjunction with an XOI, possibly due to the reduced urate production which leads to a decrease in urinary urate excretion [1]. Currently, it is only approved in combination with XOIs due to their protective effect on the kidneys [29].

A similar concern with dotinurad is warranted as a long-term observational study reported the incidence of renal calculi in five patients (1.5%) [27]. Given the similarity in mechanisms of lesinurad and dotinurad, and since hyperuricemia is a chronic condition that requires long-term treatment, careful monitoring of any possible side effects, such as renal calculi, is essential in future dotinurad treatments. In light of these findings, future studies investigating the efficacy and safety of dotinurad with concomitant XOI therapy can be anticipated. As yet, the only study that has utilized dotinurad with an XOI, topiroxostat, has shown promising results [30].

Limitations

Firstly, the studied population for all the four RCTs was restricted to the region of Japan, making it difficult to generalize the results for widespread use of dotinurad. Secondly, despite employing a random-effects model to account for the heterogeneity in the results, certain differences among the trials might complicate their interpretation. These include the use of different urate-lowering drugs as controls in the non-inferiority trials and the differences in the follow-up durations. Thirdly, aggregate data from the trials and not the patient-level data were utilized. Individual patient data is superior to aggregate data as it can uncover potential treatment correlations. Lastly, caution is advised in interpreting the safety results in this meta-analysis due to the short follow-up and lack of information on the long-term health manifestations of the intervention.

## Conclusions

Dotinurad, another addition to the arsenal of anti-hyperuricemia therapy, shows significant improvement in serum uric acid levels in hyperuricemic individuals with or without gout. Its urate-lowering effect is comparable to the commonly available antihyperuricemic agents, such as febuxostat and benzbromarone. Moreover, it is highly effective at doses 1 mg, 2 mg, and 4 mg and well-tolerated at a dose of 2 mg. Further efficacy and safety trials should be conducted to evaluate the long-term utility of dotinurad in treating hyperuricemia.
